# Combination of *ERG9* Repression and Enzyme Fusion Technology for Improved Production of Amorphadiene in *Saccharomyces cerevisiae*

**DOI:** 10.1155/2013/140469

**Published:** 2013-10-27

**Authors:** Rama Raju Baadhe, Naveen Kumar Mekala, Sreenivasa Rao Parcha, Yalavarthy Prameela Devi

**Affiliations:** ^1^Department of Biotechnology, National Institute of Technology, Warangal 506004, India; ^2^Department of Zoology, Kakatiya University, Warangal, Andhra Pradesh 506009, India

## Abstract

The yeast strain (*Saccharomyces cerevisiae*) MTCC 3157 was selected for combinatorial biosynthesis of plant sesquiterpene amorpha-4,11-diene. Our main objective was to overproduce amorpha 4-11-diene, which is a key precursor molecule of artemisinin (antimalarial drug) produced naturally in plant *Artemisia annua* through mevalonate pathway. Farnesyl diphosphate (FPP) is a common intermediate metabolite of a variety of compounds in the mevalonate pathway of yeast and leads to the production of ergosterols, dolichol and ubiquinone, and so forth. In our studies, FPP converted to amorphadiene (AD) by expressing heterologous amorphadiene synthase (*ADS*) in yeast. First, *ERG9* (squalane synthase) promoter of yeast was replaced with repressible methionine (*MET3*) promoter by using bipartite gene fusion method. Further to overcome the loss of the intermediate FPP through competitive pathways in yeast, fusion protein technology was adopted and farnesyldiphosphate synthase (*FPPS*) of yeast has been coupled with amorphadiene synthase (*ADS*) of plant origin (*Artemisia annua* L.) where amorphadiene production was improved by 2-fold (11.2 mg/L) and 4-fold (25.02 mg/L) in yeast strains YCF-002 and YCF-005 compared with control strain YCF-AD (5.5 mg/L), respectively.

## 1. Introduction 

Microbial fermentation ensures production of industrially important metabolites in large quantities. Implication of rDNA technology in microbial fermentations offers production of desired heterologous proteins at large scale. Apart from the proteins, nature offers diverse classes of complex metabolites (isoprenoids) that are utilized in the food, cosmetic, and pharmaceutical industries and so forth [1]. Many of these complex metabolites are produced naturally in low quantities in plants that are difficult or expensive to cultivate. Metabolic engineering, systems, and synthetic biology principles and methods allowed easy transfer of heterologous pathways from natural plant producer to a suitable microbial host such as yeast and *E. coli* [2–4]. *E. coli* was the most studied host for metabolic engineering of isoprenoid by modulating 1-deoxyxylulose-5-phosphate (DXP) and mevalonate (MVA) pathways [5–8]. Other studies on MVA pathway deregulation in yeasts have improved the biosynthesis of different isoprenoids [9–13]. The vital role of MVA pathway and eventual product (ergosterol) proportion from this pathway increased the interest for the engineering of isoprenoid pathway in yeast for the production of heterologous compounds [14]. Ergosterol synthesis was carried out by squalene synthase (*ERG9*), and conversion of farnesylpyrophosphate (FPP) to squalene was successfully regulated by replacing the native promoter with repressible promoters [2, 15]. ATP sulfurylase (*MET3*) catalyzes the reduction of sulfate to sulfide, involved in methionine metabolism. Addition of methionine had the ability to repress the *MET3* promoter in *S. cerevisiae* and *Ashbya gossypii *[16, 17], and *MET3* promoter was widely used as a molecular tool for yeast genetics [18]. 

Heterologous expression of pathways/enzymes in *S. cerevisiae *is complex due to the presence of numerous native host enzymes and tight regulation of the intermediate metabolites generated by the host machinery. The non native product formation is not only affected by the host environment but also by the loss of intermediate metabolites through diffusion, degradation, or converted by competitive enzymes/pathways [19, 20]. In order to avoid such intermediates loss and make heterologous expression more efficient, enzymes catalyzing successive reactions are often fused in close proximity to each other by using small linkers. Small linkers are the sequence codings for few amino acids, which separates the two proteins in space with a small distance allowing them to fold properly without constraints from each other. Consequently, the substrate was channeled between active sites of two or more sequential enzymes of a pathway, without allowing free diffusion of the intermediates. Subsequently it reducing the transit time required for the intermediates to spread the enzyme that catalyzes the next step in the reaction. Several elucidations *in vitro* and *in vivo* recommended that this strategy can be used to improve the flux through a metabolic pathway [21–25].

In the present study, we used *S. cerevisiae *(MTCC 3157) for heterologous expression of amorphadiene synthase (*ADS*) for one-step conversion of FPP to amorphadiene. In order to increase the concentration and flux of FPP towards heterologous product, squalane synthase (*ERG9*) a key enzyme in ergosterol synthesis was repressed. Further to avoid FPP loss through competitive pathways, enzyme fusion strategy was applied. Here, we composed a chimeric fusion protein between farnesyl diphosphate synthase (*FPPS*) of yeast and amorphadiene synthase (*ADS*) of *Artemisia annua *L. and was expressed in *ERG9*-repressed yeast strains. 

## 2. Materials and Methods

### 2.1. Microbial Strains, Media, and Reagents

All the reagents and media used in this study were of, analytical grade and procured either from Himedia (India) or Merck (India) or Sigma (India). The strains used in this study were *Saccharomyces cerevisiae *(MTCC 3157), and *E. coli *DH5*α* (MTCC 1652).

### 2.2. *ERG9* Promoter Replacement with *MET3* Promoter

The *MET3* promoter was amplified from genomic DNA of *S. cerevisiae* (MTCC 3157) using the primer pairs 1 and 2 (Table 1) containing restriction sites *BcuI *and *Cfr42I. *PCR was carried out in a total volume of 50 *μ*L with the following reagents: 1x *Ex Taq* DNA polymerase buffer (Stratagene), 0.2 mM dNTPs (Pharmacia), 20 pmol of each primer, and 1 U *Ex Taq* DNA Polymerase (Takara, Japan). This PCR includes two cycles of 94°C (0.5 min), 50°C (1.0 min), and 72°C (1.5 min); 29 cycles of 94°C (0.5 min), 56°C (1 min), and 72°C (1.5 min); and 72°C (5 min). Resultant PCR fragment and pUG6 plasmid (Euroscarf, Germany) were digested with *BcuI*-*Cfr42I *restriction enzymes. The vector and PCR fragments were purified with NucleoSpin gel and PCR clean-up kit (Macherey-Nagel, Germany) and separated on 1% agarose gel (Merck Biosciences, India), and gel was further purified using NucleoSpin gel and PCR clean-up kit (Macherey-Nagel, Germany). Ligation of the vector and PCR products was carried as per the standard protocol given for T_4_ DNA ligase (Merck Biosciences, India). The ligated plasmid mix was transformed into competent *E. coli *(DH5*α*) MTCC 1652, and transformants were selected on LB medium supplemented with ampicillin (50 mg/L), and the plasmid obtained was named as pUG6 *MET3*.

**Figure 1:**
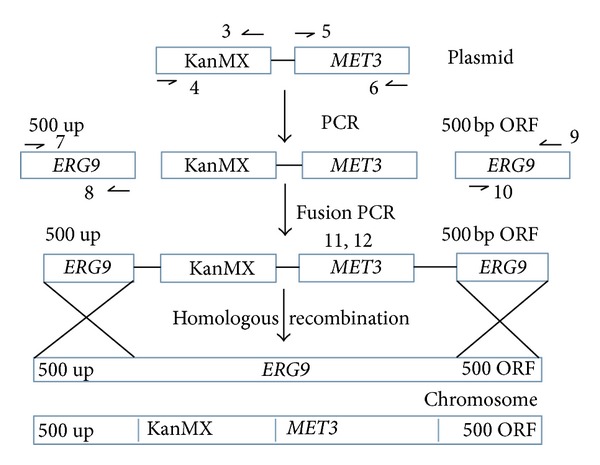
Promoter replacement of *ERG9* gene by using bipartite gene fusion method.

*ERG9 *promoter was replaced with methionine promoter by using fusion PCR, and four fragments were separately amplified before fusing them together in pairwise by using fusion PCR and a bipartite gene targeting method (Figure 1) [15, 26]. Two fragments containing the *MET3* promoter and the KanMX selection cassette were amplified from plasmid pUG6 *MET3* in two separate, but overlapping fragments using the two pairs of primers (3) a, b and (4) a, b (Table 1). Also 500 bp upstream region of the* ERG9 *promoter in the genome of *S. cerevisiae *was amplified using primers (5) and (6). Subsequently, the first 500 bp of the *ERG9* open reading frame (ORF) region was amplified using primer pairs (7) and (8) (Table 1). Finally, PCR fragments were gel purified using the NucleoSpin gel and PCR clean-up kit (Macherey-Nagel, Germany) and subsequently fused together in pairs using fusion PCR to obtain two fragments, first one with *MET3* promoter and 500 bp ORF region of *ERG9* using primers (9) and (10) and a second fragment containing KanMX and first 500 bp upstream region of *ERG9 *using primers (11) and (12) (Table 1). Subsequently, fusion PCR fragments were gel purified with the NucleoSpin gel and PCR cleanup kit (Macherey-Nagel, Germany). 

### 2.3. Construction of Fusion Proteins

An FPPS (*ERG 20*) gene fragment was obtained by PCR amplification as mentioned above using genomic DNA of *S. cerevisiae* MTCC 3157 with the primer pairs (13) and (14). The fragments were digested with EcoRI and ClaI and inserted into an EcoRI-ClaI vector fragment 2*μ* based pY01URA plasmid (Genecopeia USA) and the resulting plasmid is designated as pY01*FPPS*. For construction of plasmid expressing fusion protein *FPPS-ADS*, first *FPPS* and *ADS* were amplified separately using primer pairs (13), (14) and (15), (16) using pY01*FPPS* and pRS425ADS (Addgene#20119) as templates [2]. The two resulting PCR fragments were fused in the second round of PCR using primers (17) and (18). Similarly, *ADS-FPPS* fragments were generated in the first round using the primer pairs (19), (20) and (21), (22) and the resulting fragments fused together in second round of PCR using primer pair (23) and (24). The resulting two PCR fragments were cut with EcoRI-ClaI and individually cloned into EcoRI-ClaI plasmid 2*μ*-based pY01URA plasmid (Genecopeia, USA) and the resulting plasmids were named as pY01*FPPS-ADS *and pY01*ADS-FPPS*, respectively.

### 2.4. Strain Construction

Transformation of all strains of *S. cerevisiae* was performed by lithium acetate and PEG mediated transformation by using Yeastmaker transformation system 2 kit (Clontech, USA). *S. cerevisiae* strain (MTCC 3157), was used as the parent strain for all *S. cerevisiae* strains used in this study. Strain YCF-AD was constructed by the transformation of pESC-URA-ADS plasmid (constructed by using pESC-URA and Addgene#20119) in to MTCC 3157. Strain YCF-002 was generated by transforming the fusion PCR fragments in to strain YCF-AD and selected on Kanamycin and synthetic defined (SD)-URA drop out plates. Finally strain YCF-001 obtained by exclusion of plasmid pESC-URA-ADS from strain YCF-002 by selection on plates containing 5-fluoroorotic acid (5-FOA). Strain YCF-004, YCF-005, and YCF-006 constructed by transforming the plasmids pY01*FPPS*, pY01*FPPS-ADS, *pY01*ADS-FPPS*, and the transformants were selected on SD-URA drop out plates (Table 2).

### 2.5. OD Measurement and Dry Weight Analysis

Optical density values of samples in triplicates were measured at 600 nm (OD_600_) by using UV-Spectrophotometer (Thermo Scientific, USA). Cultures were further diluted until the OD_600_ value as <1.0 [14]. The dry weight was analyzed by using nitrocellulose filter papers (pore size 0.45 *μ*m, Whatman). The filter papers were predried in a microwave oven at 60°C for 10 min. A known volume of the cell culture was filtered, and the residue was washed with distilled water and dried in an oven at 60°C [27].

### 2.6. Ergosterol Extraction and Analysis

An overnight culture grown in minimal medium supplemented with 1.5 mM methionine (glucose 20 g/L) was centrifuged at 5,000 rpm for 5 min to get approximately 3 g of dry cells. The cell pellet was washed with distilled water, and the cell suspension was centrifuged for another 5 min at 5,000 rpm. Further cell pellet was mixed with 300 mL of 25% alcoholic KOH solution and vortexed for 1 min, and the suspension was saponified for 3 h at 90°C in a reflux. After cooling them to room temperature, nonsaponified sterols were extracted by adding 300 mL heptane followed by vortexing for 2 min. A vortex mixture of 10 mL heptane and 10 mL of alcoholic KOH solution is used as blank. After clarification of heptane layer 0.5 mL of heptane from both sample and blank was diluted tenfolds with 4.5 mL absolute ethanol. The absorbance of all samples were read against blank at 230 and 281.5 nm, respectively [14, 28].

 The ergosterol content was calculated as milligram ergosterol per gram dry weight using the following equation [28]:

(1) $$\begin{array}{c} \text{Ergosterol}=\%\;\;\text{ergosterols}-\%24\;\;\text{(}28\text{)-dehydroergosterol}\\ \text{Ergosterol }\left(\frac{\text{mg}}{\text{gDW}}\right)=\left(\frac{\text{OD}281.5}{290}-\frac{\text{OD}230}{580}\right)\times F, \end{array}$$

where *F* is a correction factor for dilutions and sample sizes, and 290 and 580 are *E* ^(1%,1 cm)^ of crystalline ergosterol and 24 (28)-dehydroergosterol, respectively.

### 2.7. Cultivation of Yeast in Shake Flask

Hundred milliliter medium was prepared with the following composition (g/L): galactose, 20; KH_2_PO_4_, 14.4; (NH_4_)_2_SO_4_, 7.5; MgSO_4_ 7H_2_O, 0.5; trace metal solution, 2; and vitamin solution, 1 mL and 50 *μ*L/L silicone antifoam. The pH was adjusted to 6.20 using 1 M NaOH and autoclaved separately from the carbon source solution. Vitamin solution was filter sterilized and aseptically added to the medium after autoclaving. Variou concentrations of methionine (0–3 mM) were used to know the minimal methionine concentration for the repression of *ERG9* expression. Flasks were further incubated in a shaking incubator (Remi, India) at 30°C with 150 rpm.

### 2.8. Batch Fermentation

Batch fermentation was carried out in a controlled bioreactor (Spectrochem, India) containing 2 L mineral medium that consists of (g/L) galactose 20; (NH_4_)_2_SO_4_, 5; KH_2_PO_4_, 3; MgSO_4_7H_2_O, 0.5; EDTA, 0.015; ZnSO_4_·7H_2_O, 0.0045; CoC1_2_·6H_2_O, 0.0003; MnC1_2_ 4H_2_O, 0.001; CuSO_4_ 5H_2_O, 0.0003; CaC1_2_·2H_2_O, 0.0000045; FeSO_4_·7H_2_O, 0.0003; NaMoO_4_,·2H_2_O, 0.0004; H_3_BO_3_, 0.001; KI, 0.0001; and 0.025 mL silicone antifoam (Merck). This medium was further autoclaved at 121°C for 20 min. Further filter sterilized vitamin solution containing (mg/L): biotin, 0.05; calcium pantothenate, 1; nicotinic acid, 1; inositol, 25; thiamine HCl, 1; pyridoxine HCl, 1; and para-aminobenzoic acid, 0.2 was added to the autoclaved mineral medium. Finally, media were supplemented with 2 mM filter sterilized methionine. During the fermentation process, the temperature was kept constant at 30 ± 2°C, and dissolved oxygen tension (50%) was maintained with sterilized air (0.2 *μ* filter) with airflow 1 L/min and with 250 rpm agitation and the off-gas passed through a outlet port. pH was controlled between 6.20 ± 0.5 by automatic addition of 1 M NaOH and 1 M HCl. Seed culture with OD_600_ of 0.02 from shake flask was inoculated into batch fermentor. After cells reaching 1 at OD_600_ 20% (vol/vol) isopropyl myristate (Merck Millipore, Germany) was added aseptically to the media. This isopropyl myristate layer was sampled and diluted with ethylacetate for determination of amorphadiene concentration by gas chromatography coupled mass spectrometry (GC-MS) (Agilent Technologies, USA).

### 2.9. Analysis of Sesquiterpenes

Amorpha-4,11-diene and farnesol were analysed by gas chromatography with flame-ionization detector (GC/FID). Samples from fermentor were centrifuged at 5000 rpm for 5 min, diluted directly into ethyl acetate, and mixed for 30 min on a vortex mixer. After phase separation 0.6 mL of the ethyl acetate, layer was transferred to a capped vial for analysis. The ethyl acetate-extracted samples were analyzed using the GC/FID. A 1 *μ*L sample was split 1 : 20 and separated using a DB-WAX column (50 m × 200 *μ*m × 0.2 *μ*m) with hydrogen as the carrier gas with flow rate of 1.57 mL/min. The temperature program for the analysis was as follows. The column was initially held at 150°C for 3.0 min, followed by a temperature gradient of 5°C per min to a temperature of 250°C. Amorpha-4,11-diene and farnesol peak areas were converted to concentration values from external standard calibrations using authentic compounds [29].

### 2.10. Expression, Purification, and SDS-PAGE Analysis of the Proteins

Different yeast strains carrying the plasmids p*ADS, *p*FPPS*, p*FPPS*-*ADS*,and p*ADS*-*FPPS *were grown in appropriate SD drop out media at 37°C. After cells optical density values were reaching 2.5 at OD_600_, they were harvested and centrifuged at 5000 rpm for 5 min, and cell pellet was resuspended in 10 mL distilled water, and proteins were extracted according to CelLytic Y Plus Kit (Sigma, USA). Purification was carried out in a single step using immobilized metal affinity chromatography (IMAC). The supernatant was applied to a 5 mL Nickel CL-Agarose Column (Merck Biosciences, India), loaded with nickel. Further nonbound proteins were removed with washing buffer. Elution of adsorbed recombinant proteins was achieved with elution buffer (Merck Biosciences, India). Fractions were collected either used or stored at 4°C. SDS/PAGE was carried out in 10% resolving and 5% stacking gels according to instructions given by the manufacturer (Merck Biosciences, India) and the samples along with protein marker were loaded on Mini-PROTEAN Tetra cell (Bio-Rad, USA). After electrophoresis, the gel was stained with coomassie blue. Gel, visualized and analyzed in Gel Doc Fire Reader Documentation System (Merck Biosciences, India).

## 3. Results

### 3.1. Effect of Methionine on *ERG9* Expression

**Figure 2:**
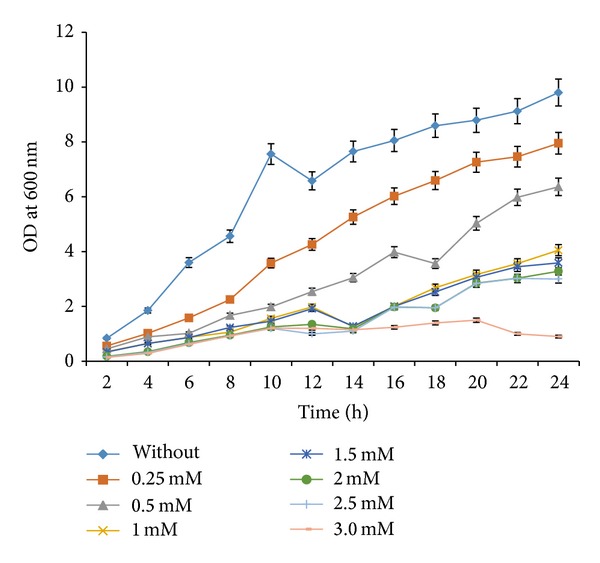
Effect of methionine on the growth pattern of *ERG9* repressed yeast strain (YCF-005) cultivated in shake flasks containing minimal medium and 20 g/L glucose (average data obtained from triplicate of the experiments were represented).

The minimum concentration of methionine for expression of *ERG9* was determined by growing the *ERG9 *repressed yeast cells in shake flasks supplemented with varying quantities of methionine (Figure 2). When yeast cells grown in the absence of methionine, cells propagated exponentially up to 10 h and followed by a slight drop in the growth and immediately after 12 h cells followed exponential phase up to 20 h. The methionine concentration at 1 mM elongated the lag phase and a lower final biomass concentration. There was a drastic decrease in the growth of the yeast beyond 2.5 mM methionine concentration, and the consequences were observed by measuring the ergosterol content (Table 3). The final ergosterol content of control yeast strain (MTCC 3157) was observed at 19.25 mg/g DW, and strain YCF-005 was given varied quantities of ergosterol at 0, 1, 2, 2.5, and 3 mM methionine as 19.22, 14.05, 13.98, and 13.75 and 3.31 mg/g DW, respectively (Table 3).

### 3.2. Combined Effect of *ERG9* Repression and Chimeric Protein Expression

**Figure 3:**
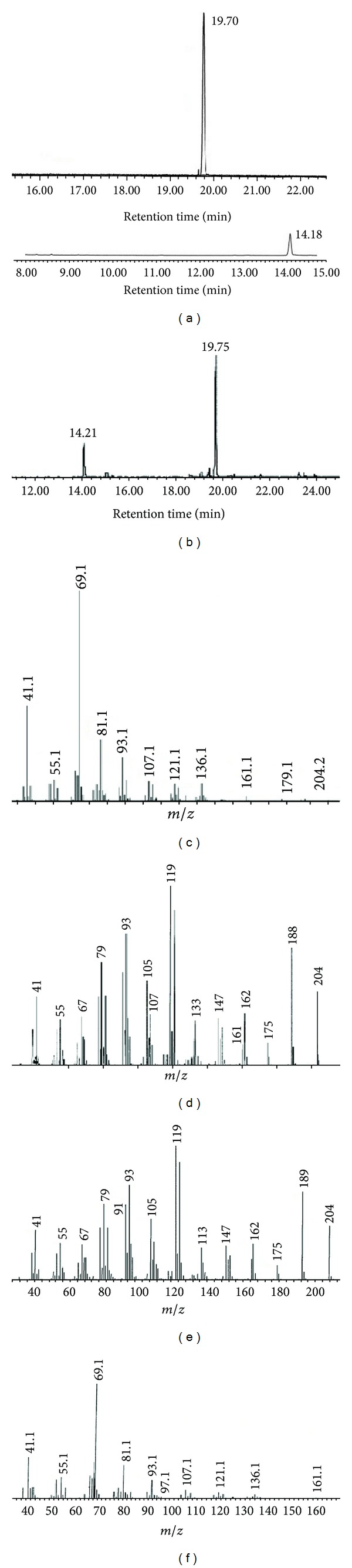
GC-MS profile of the amorphadiene and farnesol produced by YCF-005 strain. (a) Standard chromatogram of amorphadiene and farnesol, (b) ethyl extract sample chromatogram, (c) farnesol authentic standard mass spectrum, (d) amorphadiene authentic standard mass spectrum, (e) amorphadiene, and (f) farnesol sample mass spectrum generated from sample chromatogram (b).

**Figure 4:**
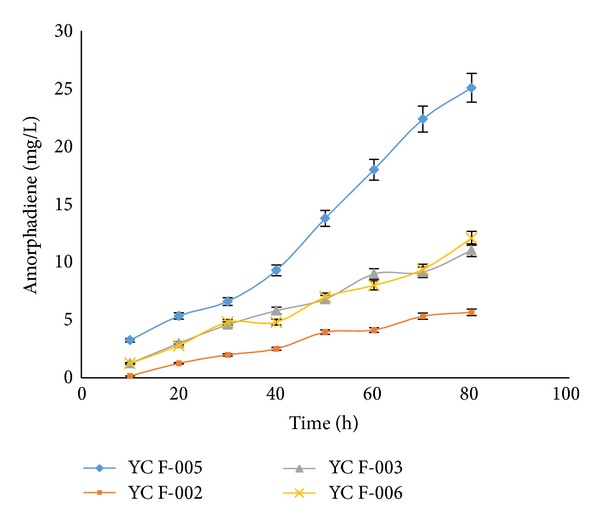
Amorphadiene concentration as a function of time in repressed (YCF-002, YCF-005, YCF-006) and nonrepressed (YCF-AD) yeast strains expression free enzymes (*ADS*) and chimera enzymes (*FPPS-ADS *and* ADS-FPPS*) (average data obtained from triplicate of the experiments were represented).

Transformed yeast strains harbouring amorphadiene synthase (ADS) was able to produce amorpha,4-11-diene (Figure 3). Strain YCF-005 analyzed for amorphadiene production by GC-MS as mentioned in materials and methods. Analysis of GC-MS for ethyl acetate extracts revealed the presence of amorphadiene major peak and other sesquiterpene as a minor peak (Figure 3(b)). Retention time, mass spectrum of main component (19.75 and 14.21 min) were almost matching with standard (19.70 and 14.18 min) (Figures 3(a), 3(c), and 3(d)). When AD producing strains analyzed for amorphadiene, strain YCF-002 produced 11.02 mg/L of amorphadiene, which was approximately 2-fold higher than AD (5.65 mg/L) produced by YCF-AD respectively (Figure 4). 

FPP conversion to farnesol in the strain YCF-004 was limited by expressing the fusion protein in yeast and to probe the effect of enzyme fusion on diversion of the flux more efficiently towards amorphadiene production. Different strains expressing the native proteins and chimeric proteins *ADS*, *FPPS-ADS*, and *ADS-FPPS* were analyzed. The strain transformed with the plasmid expressing ADS-FPPS produced amorphadiene (12.08 mg/L) at the same level as that for the strain expressing free *ADS* (Figure 4). 

More interestingly, the strain transformed with the plasmid expressing *FPPS-ADS* produced amorphadiene at an almost 2-fold higher level (25.06 mg/L) than that for the strain expressing free *FPPS* and *ADS* (Figure 4). 

### 3.3. SDS-PAGE Analysis

**Figure 5:**
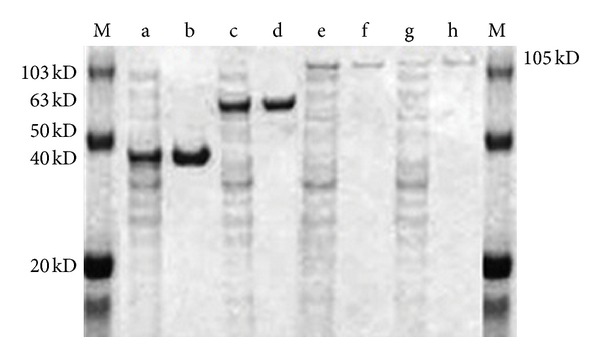
SDS/PAGE of recombinant enzymes produced in different yeast strains. Lane M: molecular mass standards; lane a, crude extract FPPS; lane b: purified FPPS; lane c: crude extract ADS; lane d: purified ADS; lane e: crude extract FPPS-ADS; lane f: purified FPPS-ADS lane g: crude extract ADS-FPPS; lane h: purified ADS-FPPS. The calculated molecular weights of FPPS, ADS, and the fusion enzymes were 40, 63, and 103 kDa, respectively.

The four recombinant proteins were produced using the conditions reported as above. On SDS-PAGE the four proteins were compared across the protein molecular marker (Figure 5). Both fused proteins *FPPS-ADS* and *ADS-FPPS* molecular weights were approximately 105 kD which was almost equal to the sum of two individual proteins (FPPS 40 kD and 63 kD). This indicates the efficacious fusion of the two proteins to form a chimeric protein.

## 4. Discussion

In the present study, we have improved the amorphadiene production by 4-fold in the yeast *S. cerevisiae* (MTCC 3157). In order to decrease the FPP flux towards ergosterol and competitive pathways, *ERG9* promoter was replaced with repressible methionine (*MET3*) promoter, and strain YCF-005 growth rate was very low at 3.0 mM methionine concentration, due to low ergosterol production. There was no much effect on the growth beyond 1.5 mM methionine. One and half mM methionine was selected for the regulation of the *MET3 *promoter. The ergosterol content of the *ERG9* repressed strain was quite low compared with control strain (MTCC 3157). Increasing concentration of methionine repressed the yeast strain and produced varied quantities of ergosterol (Table 3) upon increasing methionine, there was a tight regulation of the promoter. Subsequent repression of *ERG9* leads to the accumulation of FPP derived farnesol. Farnesol accumulation observed in earlier studies with deletion and inhibition of *ERG9* gene [30–32]. Moreover farnesol at low concentration inhibits the growth of the cells by cell cycle arrest in some yeast cells [33]. We observed that strain YCF-001 produced 12.03 mg/g DW of farnesol and final concentration 24.12 mg/L and disclosed the possibility of pooling the available FPP towards amorphadiene by enzyme fusion technology. As mentioned in earlier studies, a discrepancy of methionine was not sufficient to repress *ERG9* during fermentation and 2.5 mM methionine was maintained during fermentation [15]. 

We used isopropyl myristate as a solvent for trapping the volatile compounds and for phase separation, and that phase was analyzed for determination amorphadiene by GC-MS. Analysis of ethyl acetate extracts revealed the capability of yeast strains carrying *ADS* gene for production of amorphadiene. The major peak in the chromatogram confirmed as a amorphadiene and other minor peak as a farnesol with corresponding standards (Figures 3(a) and 3(b)) [34, 35]. *ERG9* (YCF-002) repression improved the amorphadiene production by increasing the FPP availability when compared with amorphadiene production in strain YCF-AD. Strains YCF-AD and YCF-002 were cultured in the fermentor and analyzed for amorphadiene and farnesol. Strain YCF-AD produced 5.65 mg/L of amorphadiene and 4.12 mg/L farnesol, as there was a 2-fold (11.02 mg/L) rise in amorphadiene production in strain YCF-002 (Figure 4). To overcome the natural loss of the metabolic intermediate FPP and its conversion towards farnesol, a chimeric protein with small Gly-Ser-Gly linker was constructed as *ADS-FPPS* and *FPPS-ADS*. Strain YCF-006 expressing *ADS-FPPS* produced 12.08 mg/L of amorphadiene which was almost close to amorphadiene produced by strain YCF-002 as YCF-005 produced 25.06 mg/L of amorphadiene and which was 2-fold higher than than the YCF-002 and subsequently 5-fold higher than YCF-001 (Figure 4). Strains (YCF-005) expressing *FPPS-ADS* do not produce higher amorphadiene yields compared with strains YCF-005 and YCF-002 as the active sites were likely far apart to produce a beneficial proximity effect [13]. The fusion phenomenon of the enzyme further confirmed by isolation and purification of *FPPS*, *ADS*, *ADS-FPPS*, and *FPPS-ADS* enzymes with SDS-PAGE and fused proteins molecular weights (105 kD) were almost equal to the sum of the individual enzymes *ADS *(63 kD) and *FPPS* (40 kD) (Figure 5). 

## 5. Conclusion

Enzyme fusion technology will be an additive step to the metabolic engineering, and combination of metabolic engineering and enzyme fusion technology improves the flux of the intermediate metabolites in order to expand the heterologous components production. Further combinatorial expression of *tHMGR* and upregulation of *UPC2 alleles* with fusion technology accelerate the amorphadiene production. Further channeling acetyl-CoA into the mevalonate pathway will make it possible to further increase the FPP production which is having tremendous significance in chemical industry as well as in medicine.

## Figures and Tables

**Table 1:** List of the primers used in this study.

List of primers
(1) GGGACTAGTGTTTAATTTAGTACTAACAGAGACTT
(2) CCCCCGCGGCATGTTAATTATACTTTATTCTTGTT
(3) GATCCCCGGGAATTGCCATGACGCTGCAGGTCGACAACCC
(4) CCATGAGTGACGACTGAATCCGG
(5) CTATCGATTGTATGGGAAGCCCG
(6) CAATGCCAATTGTAATAGCTTCCCAT
(7) GTTAATTATACTTTATTCTTGTTATTATTATAC
(8) AGCCTCAGTACGCTGGTACCCG
(9) CATGGCAATTCCCGGGGATCTGGGCTATGAAATGTACTGAGTCAG
(10) ATGGGAAAGCTATTACAATTGGCATTG
(11) GTCGTA-GTCGTGGACGGTTTGC
(12) AGCCTCAGTACGCTGGTACCCG
(13) ATGGCTTCAGAAAAAGAAATTAGGAGAGAGAGA
(14) TTCAGTCAAGGCCACTATTTGCTTCTCTTGTAAACTTTGTTCAAGAAC
(15) GAGAAGCAAATAGTGGCCTTGACTGAAGAGAAACCTATAAGGC
(16) TTAGATAGACATAGGGTAAACTAGCAATGATTTGATCAA
(17) ATGGCTTCAGAAAAAGAAATTAGGAGAGAGAGA
(18) TTAGATAGACATAGGGTAAACTAGCAATGATTTGATCAA
(19) ATGGCCTTGACTGAAGAGAAACCT
(20) ATTTCTTTTTCTGAAGCCATTTAGATAGACATAGGGTAAACTAGCAATGATTTG ATCAA
(21) TTTACCCTATGTCTATCTAAATGGCTTCAGAAAAAGAAATTAGGAGAGAGAG
(22) CTATTTGCTTCTCTTGTAAACTTTGTTCAAGAACG
(23) ATGGCCTTGACTGAAGAGAAACCT
(24) CTATTTGCTTCTCTTGTAAACTTTGTTCAAGAACG

**Table 2:** Yeast strains and plasmids used in this study.

Strain	Genotype	Plasmid	Reference
MTCC 3157	*MAT*α* his3Δ1 leu2Δ0 lys2Δ0 ura3Δ0 *	None	Imtech
YCF-001	*MAT*α* his3Δ1 leu2Δ0 lys2Δ0 ura3Δ0, erg9::pMET3-ERG9 *	None	In this study
YCF-AD	*MAT*α* his3Δ1 leu2Δ0 lys2Δ0 ura3Δ0 2*µ* pESC-URA-ADS *	*pESC-URA-ADS *	In this study
YCF-002	*MAT*α* his3Δ1 leu2Δ0 lys2Δ0 ura3Δ0, erg9::pMET3-ERG9 pESC-URA-ADS *	*pESC-URA-ADS *	In this study
YCF-004	*MAT*α* his3Δ1 leu2Δ0 lys2Δ0 ura3Δ0, FPPS *	pY01*FPPS *	In this study
YCF-005	*MAT*α* his3Δ1 leu2Δ0 lys2Δ0 ura3Δ0, erg9::pMET3-ERG9 FPPS-ADS *	pY01*FPPS-ADS *	In this study
YCF-006	*MAT*α* his3Δ1 leu2Δ0 lys2Δ0 ura3Δ0, erg9::pMET3-ERG9 ADS-FPPS *	pY01*ADS-FPPS *	In this study

**Table 3:** The final ergosterol content with varying concentrations of methionine (average data obtained from triplicate of the experiments were represented).

Strain	Methionine concentration (mM)	Ergosterol (mg/g DW)
MTCC 3157	0	19.25
YCF-005	0	19.21
YCF-005	1	14.05
YCF-005	1.5	13.98
YCF-005	2.0	13.75
YCF-005	2.5	12.57
YCF-005	3.0	3.31
